# End-Point Variability Is Not Noise in Saccade Adaptation

**DOI:** 10.1371/journal.pone.0059731

**Published:** 2013-03-21

**Authors:** James P. Herman, C. Phillip Cloud, Josh Wallman

**Affiliations:** 1 Department of Biology, The City College of New York, New York, New York, United States of America; 2 PhD Program in Biology, The Graduate Center, The City University of New York, New York, NY, United States of America; 3 PhD Program in Psychology, The Graduate Center, The City University of New York, New York, New York, United States of America; University of Muenster, Germany

## Abstract

When each of many saccades is made to overshoot its target, amplitude gradually decreases in a form of motor learning called saccade adaptation. Overshoot is induced experimentally by a secondary, backwards intrasaccadic target step (ISS) triggered by the primary saccade. Surprisingly, however, no study has compared the effectiveness of different sizes of ISS in driving adaptation by systematically varying ISS amplitude across different sessions. Additionally, very few studies have examined the feasibility of adaptation with relatively small ISSs. In order to best understand saccade adaptation at a fundamental level, we addressed these two points in an experiment using a range of small, fixed ISS values (from 0° to 1° after a 10° primary target step). We found that significant adaptation occurred across subjects with an ISS as small as 0.25°. Interestingly, though only adaptation in response to 0.25° ISSs appeared to be complete (the magnitude of change in saccade amplitude was comparable to size of the ISS), further analysis revealed that a comparable proportion of the ISS was compensated for across conditions. Finally, we found that ISS size alone was sufficient to explain the magnitude of adaptation we observed; additional factors did not significantly improve explanatory power. Overall, our findings suggest that current assumptions regarding the computation of saccadic error may need to be revisited.

## Introduction

Because a small (roughly 1° in diameter), central region of the human retina (the fovea) has the greatest receptor density, the best view of an environmental stimulus is achieved by accurately orienting gaze towards it. We move our gaze from point-to-point using rapid movements called saccades. Somewhat surprisingly, the accuracy of saccades changes little with age [1], [2], suggesting that movement accuracy is actively monitored and maintained. Indeed, experiments on humans with extraocular muscle paresis [3]–[5] and tenectomized monkeys [6], [7] demonstrated an impressive endogenous ability to restore normal saccade amplitudes in a relatively brief period (on the order of days). Meanwhile, *McLaughlin* noted that modification of saccade amplitude can be achieved by an intrasaccadic step (ISS) paradigm, applying experimentally arranged, primary-saccade-triggered secondary target shifts in a series of successive saccade trials [8].

This maintenance, restoration, or manipulation of saccade size is termed “saccade adaptation.” Since it was recognized that the adaptive changes achieved by the ISS paradigm are the same as those resulting from tenectomy [9], the majority of adaptation studies have relied on the ISS paradigm to extensively explore and document the multiplicity of subtle ways that saccade adaptation can display sensitivity [10], [11].

There are two major classes of ISS paradigm. The first is identical to the method used by *McLaughlin*: the amplitude of the ISS is fixed (in degrees of visual angle, or as a percentage of the primary target step) [8]. The second is a variant that reflects the increasingly widespread belief that saccade adaptation is driven by retinal error (the difference between gaze and target position): the ISS is chosen on each trial to ensure that the primary saccade results in a specific retinal error. Though experiments of the first class are by far more common [10], [11], only experiments of the second class have been used to address such basic issues as the adaptive response to a range of error sizes [12], [13] or to errors with added noise [14], or the possibility that adaptation is driven by the violation of error prediction rather than pure retinal error [15]. The one study (of which we are aware) that compared the effects of different ISSs used only 2 subjects, and 2 ISS values (25% & 50%), making their results somewhat difficult to interpret [16]. Thus, a systematic exploration of the effects of varying ISS-size has never been undertaken.

To address this issue, our current experiments characterized gain-decrease adaptation in response to a variety of fixed ISS amplitudes (where gain is defined as the ratio of saccade amplitude to primary-target-step size). We chose to only conduct gain-decrease experiments because gain-increase adaptation has been shown to have distinct characteristics [9], [16]–[21], and has been suggested to employ distinct mechanisms [22]–[24]. We used ISSs with amplitudes of 1°, 0.75°, 0.5°, 0.25°, 0.1° and 0° (as a control), following 10° primary target steps (0.1, 0.075, 0.05, 0.025, 0.01, and 0.0 in gain-units). Our primary goal was to find the smallest ISS effective for inducing significant adaptation. We were also curious if saccade adaptation, like other forms of motor learning [25], [26], would respond differently to smaller errors. Lastly, we wondered whether any inter-subject differences in adaptation magnitude might be related to aspects of baseline (pre-adaptation) saccade metrics (such as end-point variability or undershoot) as was the case in a fixed-retinal-error ISS paradigm [15], and as has been implied in modeling efforts [27].

## Methods

### General

Subjects were instructed prior to each session that they would be presented with a small red annulus that would begin in each trial on the left, step to the right at a random time, and that they should track it with their gaze.

Subsequent to receiving instructions, each participant sat in a darkened room, 57 cm from the display, and underwent a nine-point self-paced calibration prior to the start of recording.

### Stimuli and Procedure

The fixation and target stimulus was a small (0.3°) red annulus, on a dark background.

All target positions were determined prior to all recordings and were identical for each subject.

**Figure 1:**
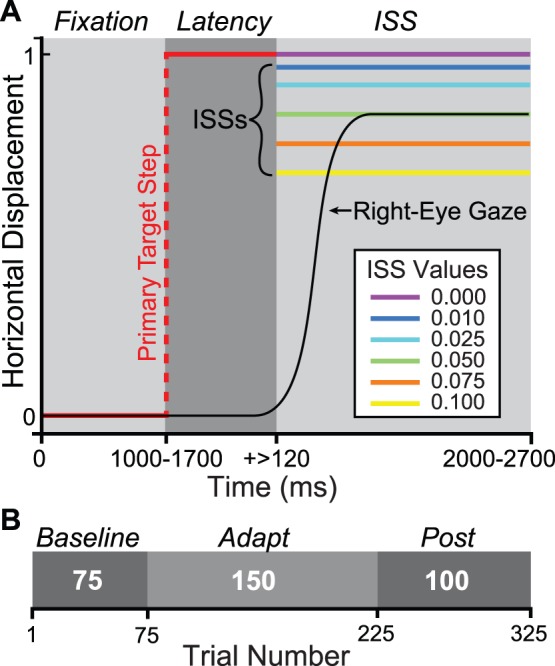
Methods. A. Single-Trial Temporal & Spatial Structure. Red: pre-saccade target; black: right-eye gaze; violet: 0.0 (no) ISS; blue: 0.01; light blue: 0.025; green: 0.05; orange: 0.075; yellow: 0.1. ISSs and primary target step are not drawn to scale. Gray scale boxes and italic text above indicate trial temporal windows. Fixation: pre-target-step fixation period; Latency: delay (ms) between primary target step and primary saccade onset; ISS: intrasaccadic target step and subsequent corrective saccade period. B. Experimental session phase structure. Phases are depicted as gray scale boxes with phase names above (italic). White numerals in each box indicate phase-length in trials. Absolute trial number is indicated on the axis.

We used 2 trial-types: (1) no-ISS and (2) ISS trials (Figure 1A). In no-ISS trials, the subject fixated the target on the left portion of the display (initial position drawn uniformly from the interval [−7.5°, −2.5°]) for a random period (drawn uniformly from: [1000 ms, 1700 ms]). The target stepped (amplitude uniformly drawn from [9.5°, 10.5°]), and remained in place until the end of the trial (total trial duration ranged from 2000–2700 ms: Total Duration = 1000 ms+Fixation Duration). Meanwhile, in ISS trials, the only difference was that upon detection of the subject’s primary saccade (velocity >25°/s), the target stepped intrasaccadically by a value depending on the condition (Figure 1A). Possible values (in gain-units) were 0.1, 0.075, 0.05, 0.025, 0.01 and 0.0, thus in the 0.0-ISS condition, ISS trials were identical to no-ISS trials. Other than the 0.0 condition, which was added as a control after feedback from preliminary data presentation at a conference, each subject was exposed to each condition in a random order, and we always allowed several days to elapse between sessions in order to minimize the likelihood that learning was retained between sessions.

Each experimental session comprised 3 phases, a 75-trial baseline consisting exclusively of no-ISS trials, a 150-trial adapt phase of ISS trials, and a 100-trial post or recovery phase of no-ISS trials (Figure 1B).

### Ethics Statement

Informed written consent was obtained prior to any recording sessions, and protocols were reviewed and approved by the Institutional Review Board (IRB) of the City College of New York (CCNY), and thus complied with all human-subject protocol requirements.

### Subjects

Data was collected from 6 subjects, ages 25–37, 2 female and 4 male. All were faculty and students from the CCNY community and had normal or corrected-to-normal vision. Of these subjects, 2 were authors (JH and CC), and 3 were naïve to the purposes of the experiment. We found no meaningful differences between naïve and non-naïve subjects.

### Equipment

Stimuli were displayed on an Iiyama Vision Master Pro 514 CRT display (Oude Meer, Netherlands) at a resolution of 800×600 pixels (visible area 41.5 cm×30.5 cm), and a vertical sync-rate of 200 Hz with 8-bit color depth.

Stimulus generation and display, data storage, and overall experimental session orchestration were controlled with a custom interface in LabView (National Instruments, Austin, TX) running in Windows XP (Microsoft Corporation, Redmond, WA) on a Dell PC (Austin, TX).

Eye movements and gaze position were measured and collected by an Eyelink-1000 infrared camera system (SR-Research, Mississagua, Ontario, Canada), which sampled right-eye gaze (pupil - corneal reflection) at 1000 Hz, with a spatial resolution of 0.01°.

### Analysis and Statistics

Data were analyzed using a purpose-written interface in Matlab (The Mathworks Inc., Natick, MA). Saccades were first detected automatically using a 10°/s velocity threshold, and confirmed by visual inspection. A small number of trials (<5%) were discarded due to blinks or hypometric primary movements (<50% of target eccentricity).

**Figure 2:**
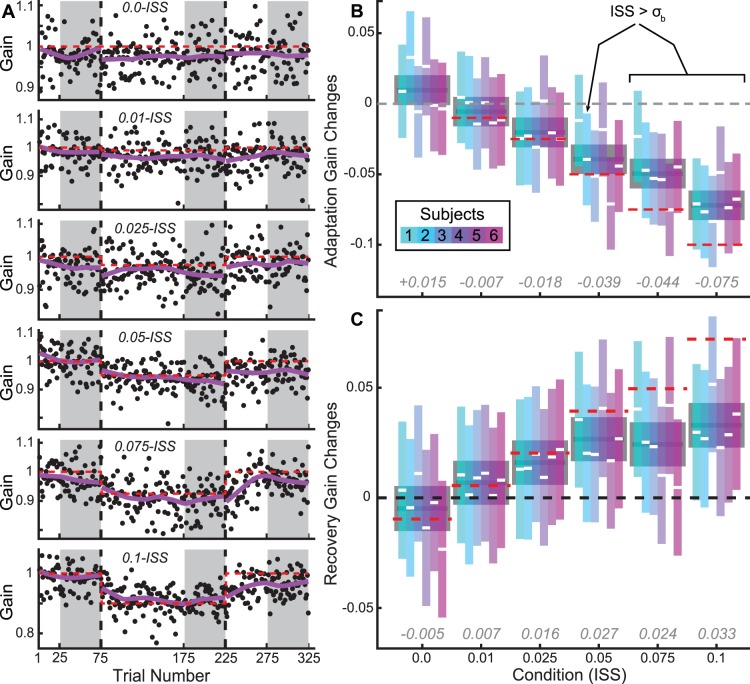
Gain adaptation and recovery. A. Raw data from an example subject. Primary saccade gains (black dots) are plotted versus trial numbers for each ISS condition, with 0.0-ISS at top, down to 0.1 at bottom. Red dashed lines indicate the final target position, and purple traces are robust lowess smooths, this color indicates the example subject’s data, also in purple, at right (subject 5), in B and C. Gray shaded regions indicate those used to calculate adaptation and recovery magnitudes plotted in B, and C. For the details of this procedure, see Methods. Briefly, adaptation was calculated by the difference between the means of the final 50 trials of baseline and adapt phase using the Tukey-Kramer method after an ANOVA. B. Magnitude of adaptation across and within subjects. Gray boxes and dark gray line segments in background represent across-subjects adaptation, while colored boxes and white line-segments represent individual subject adaptation (mean ±95% CI: Confidence Interval; box colors correspond to subjects as indicated in legend). Dashed grey line represents 0 adaptation, and may be used to determine significance by comparison with group or individual CIs (non-overlapping meaning significant adaptation). Red dashed lines represent ISS values (in gain units), and grey italic text above axis indicates mean across-subjects adaptation magnitude. We have also indicated those cases in which the ISS value was greater than the subject’s baseline variability (σ_b_) in that session. C. Magnitude of recovery across and within subjects. Conventions as in B, except red dashed line-segments now indicate (sign-reversed) adaptation magnitude as calculated above (in A) and grey italic text above axis now indicates the magnitude of recovery across subjects. Black dashed line represents 0 recovery, and may again be used to determine significance by comparison with a CI of interest.

We quantified gain changes (adaptation and recovery) starting with a repeated-measures Analysis of Variance (ANOVA). We then used the Tukey-Kramer method to find the differences (and associated 95% confidence intervals) between all estimated population marginal means (PMMs). For example, in determining the amount of induced adaptation and recovery amongst conditions we began with a 3×6×6 (Phase×Condition×Subject) ANOVA; the three levels in the Phase factor (each also corresponding to a PMM) were: (1) the final 50 trials of the baseline phase, (2) the final 50 trials of the adapt phase, and (3) the final 50 trials of the recovery phase (these are indicated by the shaded areas in Figure 2A). We then used Tukey-Kramer to compute the PMM differences: (2) – (1) for adaptation, and (3) – (2) for recovery, across and within subjects for each condition simultaneously (Figure 2B, 2C).

Where applicable, we quote test statistics and p-values. However, because the Tukey-Kramer method provides simultaneous confidence intervals (CIs) on all differences amongst PMMs (for a given α), but only provides a boundary on p-values, when we mention the statistical significance of such differences we specify only the α-value. An example would be comparing the amount of adaptation in two conditions: a comparison of two Tukey-Kramer PMM differences (end-of-baseline minus end-of-adapt in each condition) whose significance at the chosen α is ensured when the 95% CIs are non-overlapping; it is in cases of this type (when a p-value cannot be directly computed) that we provide only the α-value.

**Figure 3:**
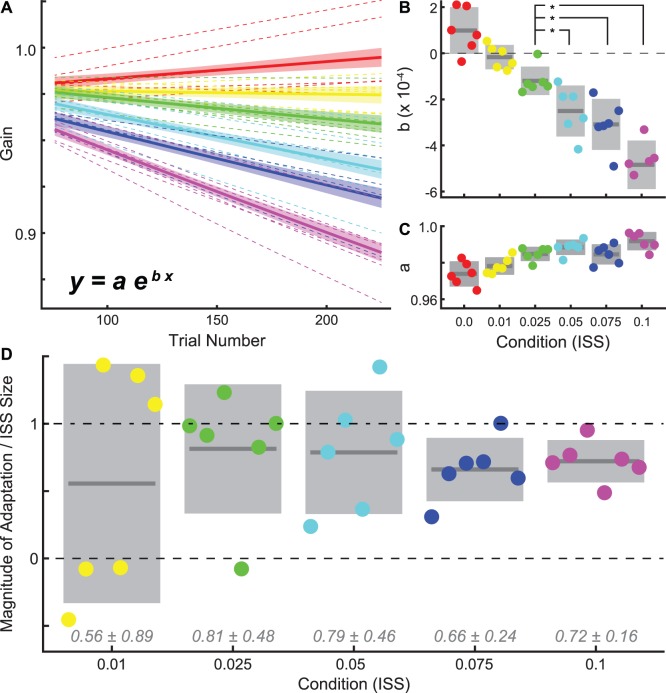
Rate of adaptation and ISS proportion learned. A. Single exponential fits to adapt-phase gains (y) as a function of trial number (x). We show fits to individual subject gains (dashed lines) as well as to gains pooled by condition (solid lines; shaded areas are 95% prediction bounds). To fit pooled data, we first additively aligned individual subject gains: setting each subject’s final 50 baseline trials to a mean across subjects. Individual conditions are colored as in B, C, and D. B. Fit rate parameter (“b”) estimates by condition (ISS). Colored dots are individual subjects, dark line segments and grey boxes are across-subject means and 95% CIs; dashed black line indicates a rate of 0. The small scale of these parameter values reflects the choice of gain and trial number as units for fitting. Asterisks indicate significant differences across subjects in ANOVA post-hoc tests at α = 0.05. C. Estimates of scale parameter (“a”) by condition; conventions as in B. D. Magnitude of adaptation expressed as a proportion of the ISS used. Colored dots again represent individual subjects while dark line segments and grey boxes means and standard errors. Dashed and dashed-dotted black lines indicate proportions of 0 and 1, respectively. Grey italic text above axis indicate mean proportion of adaptation ±95% CI across subjects.

Fitting single exponential functions (y = ae^bx^) to gain vs. trial number data was performed with the Curve Fitting Toolbox in Matlab using a least-squares approach (Figure 3). For fitting across subjects in a given condition, individual subject data were first additively normalized to align the means of each subject’s final 50 baseline trials to a mean across subjects.

Stepwise linear regression was computed using the Statistics Toolbox in Matlab.

**Figure 4:**
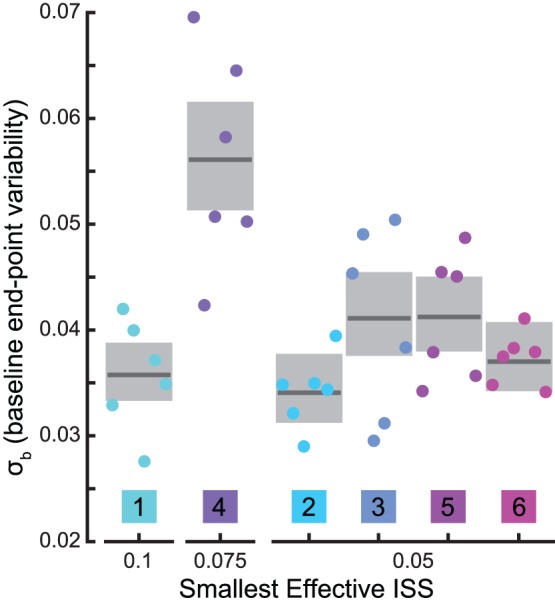
End-point variability and smallest effective ISS. We define baseline end-point variability (σ_b_) as: the standard deviation of primary saccade gain during the final 50 trials of the baseline phase. Colored dots are σ_b_ calculated for each subject in each condition; dark line segments and grey boxes are across-condition estimates and bootstrapped 95% CIs for each subject (see Methods for details). The smallest effective ISS is the minimum ISS size for which a particular subject showed a significant adaptive decrease in gain (see Figure 2A). Colors and subject numbers as in Figure 2.

The standard deviation of baseline-phase saccade endpoint, σ_b_, was calculated in two ways (1) For subject-by-subject scale comparison with a given condition’s ISS, σ_b_ was simply the standard deviation of the endpoints of the primary saccades in the final 50 trials of a given subject’s baseline phase in that condition. (2) For an across session estimate for each subject (grey boxes in Figure 4), endpoints in the final 50 baseline trials were pooled after additively normalizing them to a common mean value. These normalized and pooled data were used to compute σ_b_ again as a simple standard deviation. Meanwhile the associated confidence interval (CI) was computed by a bootstrapping procedure: 2000 samples of 50 values were drawn (with replacement) at random from the pooled data; the standard deviation of each sample was computed, yielding a distribution comprising 2000 values; the CI was then calculated as the interval containing the central 95% of this distribution.

## Results

We measured the propensity of a group of subjects to decrease their primary saccade gain (ratio of saccade amplitude to target step-size) in response to a set of fixed intrasaccadic steps (ISSs) of a point-like target. We sought the smallest ISS that was effective in causing significant adaptation both across and within subjects. We were also curious about any differences in this minimum at the subject level: might such differences be related to simple observables such as variability in landing position or the average undershoot of saccades prior to any adaptation? We asked whether subjects responded differently to smaller errors, and, finally, we attempted to determine what potential cause best explained the pattern of adaptive changes we observed.

### Changes in Saccade Metrics and Corrective Saccade Production

Relative to the 0.0-ISS control condition, adaptation caused no significant change in any saccade parameter other than gain. We found no changes in saccade peak velocity, duration, or latency that were significantly different from that of the control condition (α = 0.05).

Secondary corrective saccades occurred at an equivalent rate in the baseline and adapt phases, and at a higher rate during the recovery phase. Correctives occurred on 52.5% (±1.9; binomial parameter 95% confidence interval, CI) of baseline trials, 51.2% (±1.3) of adapt trials, and 60.5% (±1.6) of recovery trials; the difference in rate between baseline and adapt phases was not significant (χ^2^ Proportion Test, χ^2^ = 80.3, Prob>χ^2^ = 0.25) while the difference in rate between both baseline and recovery (χ^2^ = 39.8, Prob>χ^2^<<0.01) and adapt and recovery were (χ^2^ = 75.8, Prob>χ^2^<<0.01).

### Gain Changes

Across subjects, adaptation occurred with ISSs as small as 0.025, and displayed a clear trend towards greater adaptation with increasing ISS (Figure 2B). To determine the magnitude and significance of adaptation, we calculated differences in gain between the ends of the baseline and adapt phases (see Methods for details). Comparing group level CIs to 0, it is apparent that significant adaptation occurred for all ISS amplitudes greater than 0.1° (Figure 2B; α = 0.05). Interestingly, comparing CIs to ISS values (red dashed lines in Figure 2B) indicates that while adaptation was (on average) complete (indistinguishable from ISS value) in the 0.025-ISS condition (α = 0.05), larger ISSs yielded incomplete adaptation (Figure 2B; α = 0.05).

Adaptation rates, estimated by fitting, showed the same trend (with respect to ISS size) as did adaptation magnitude. We fit relationships between gain and trial number using single-exponential functions, both individually and by condition (Figure 3A; for details, see Methods). Across subjects, rate varied significantly with condition, and parameters were significantly below 0 for ISS values of 0.025 and above (α = 0.05, ANOVA, F = 13.9, Prob>F <<0.01; Figure 3B). Somewhat suprisingly, the 0.025-ISS condition had a significantly smaller rate parameter than conditions with larger ISSs (α = 0.05; Figure 3B).

Scale parameters, meanwhile, generally increased with ISS (Figure 3C). The effect of condition on variations in this parameter was significant (ANOVA, F = 2.62, Prob>F = 0.04; Figure 3C), which is unsurprising given the increasing magnitude of adaptation with ISS.

In summary, adaptation magnitude and rate were both significant in conditions with ISSs larger than 0.01. Adaptation in the 0.025-ISS condition was complete, unlike larger ISS values; but adaptation rate in that condition was smaller than those in larger ISS conditions. In order to resolve these seemingly inconsistent findings, we further examined the extent of adaptation in each condition.

### Adaptation Completeness

To better understand why adaptation appeared to be complete only in the 0.025-ISS condition, we expressed adaptation magnitude as a relative proportion of the imposed ISS rather than as an absolute change in gain-units. We calculated this proportion by dividing each subject’s adaptation magnitude in each condition (Figure 2B, white line-segments) by the ISS in that condition (Figure 2B, red dashed lines). Excluding the 0.0-ISS control, across-subject averages and CIs, along with individual subject means are plotted in Figure 3D. Fascinatingly, this analysis revealed that the extent of adaptation (as a proportion of ISS) was indistinguishable amongst conditions (ANOVA, F = 0.27, Prob>F = 0.89). This finding suggests that the completeness of adaptation observed exclusively in the 0.025-ISS condition was not the result of greater adaptation in response to smaller ISSs; rather that it results from the coincidental matching of variability with percent completeness. That is, the 81% completeness in the 0.025-ISS condition leaves ∼0.005-ISS; since the variability in adaptation magnitude (across subjects) is greater than this (0.01), the adaptation appears complete. Whereas, the 79% completeness in the 0.05-ISS leaves 0.011-ISS; placing the adaptation just shy of complete since the variability was slightly smaller (again 0.01 across subjects).

### Minimal Effective ISS and End-point Variability

Another feature of our adaptation results that deserves further consideration is the fact (apparent in Figure 2B) that individuals varied in their responsiveness to different ISSs. Most subjects showed significant adaptation in response to 0.05 ISSs (Figure 2B; α = 0.05). However, subject 4 required an ISS of at least 0.075, and the smallest effective ISS for subject 1 was 0.1 (Figure 2B; α = 0.05). In light of the foregoing analysis contrasting adaptation magnitude and proportion, it is important to note that the two were in agreement regarding the smallest effective ISS values described above.

The propensity for an individual subject to adapt was unrelated to end-point variability. When ISS>σ_b_ (standard deviation of landing position in the final 50 trials of the baseline for a given subject in a given session), significant adaptation always occurred (Figure 2B, as indicated; α = 0.05). However, when ISS<σ_b_ significant adaptation still sometimes occurred: in the 0.05-ISS condition, subjects 3, 5, and 6 displayed significant adaptation (σ_b_ = 0.051, 0.061 and 0.065, respectively), while 1 and 4 did not (σ_b_ = 0.07 and 0.058). This suggests that end-point variability is not equivalent to target perturbation sensitivity.

In order to reveal any trend relating the threshold ISS value to end-point variability, we plotted an estimate of each subject’s variability grouped by threshold (Figure 4). For an across-conditions estimate of σ_b_, we pooled each subject’s data and used a bootstrapping procedure to estimate the CI (see Methods). Interestingly, while subject 4 had the largest end-point variability across conditions (0.056) and a higher threshold for adaptation (0.075), the highest threshold (0.1) was displayed by subject 1, who had the 2^nd^ smallest end-point variability (0.035). The finding that ISS threshold was not clearly related to end-point variability further strengthens our contention that end-point variability does not mirror ISS sensitivity.

As a final component of this argument, and to explore the question of what does account for adaptive changes in gain, we included end-point variability along with several other putative predictors of adaptation in a regression analysis.

### What Best Explains Overall Adaptation Results

**Figure 5:**
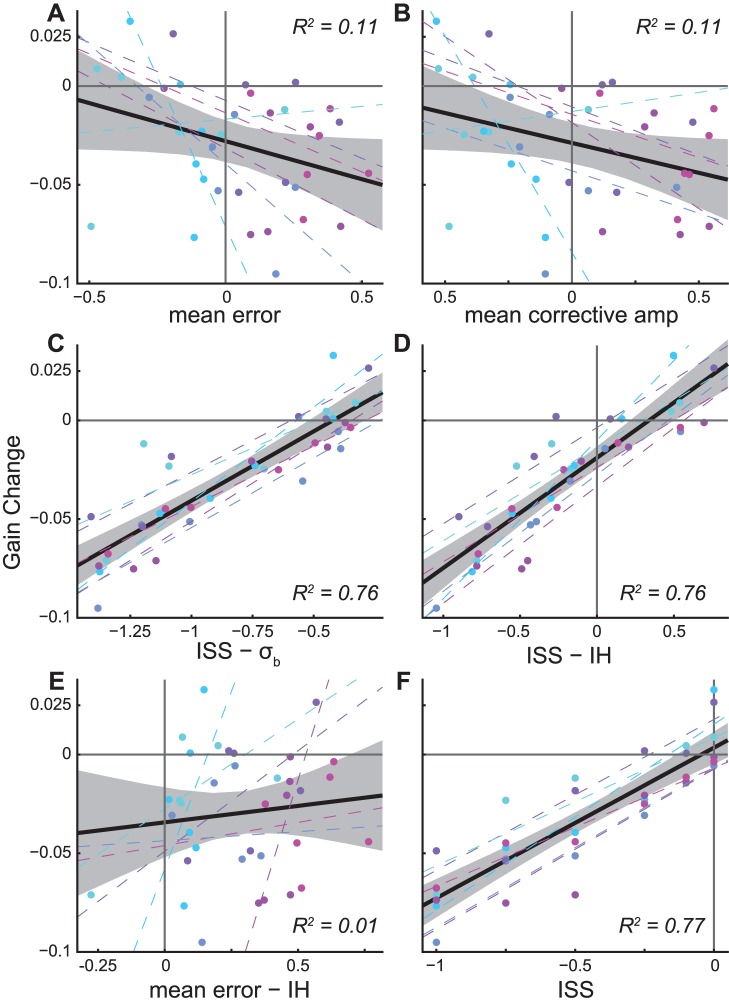
Regression analysis. In all panels, one data point (colored dots) for each subject in each condition (colors as in Figures 2, 4); dashed lines are linear least-squares fits by subject; solid black line and shaded area are linear least-squares fits across subjects and 95% prediction bounds, corresponding R^2^ values are inset; solid gray lines are x and y = 0, respectively; vertical axis is the mean amount of adaptation (for details of this calculation, see Methods). A. Mean error was calculated using the first 25 trials of adaptation, since error will be most distinct for a given condition in this initial portion of the adaptation phase; see text for clarification. B. Similarly, mean corrective saccade amplitude was calculated only in the first 25 adapt-phase trials. C. The difference between “inherent hypometria” (IH) and baseline end-point variability (σ_b_). IH was calculated as the mean undershoot during the final 50 trials of the baseline phase, the same trials were used to calculate baseline σ_b_. D. The difference between ISS and IH. E. The difference between “mean error” and IH. F. Intrasaccadic Step Size in degrees. Note that in this figure, we have chosen to use degrees of visual angle for the abscissa and gain-units for the ordinate.

ISSs best explained the collective pattern of adaptation observed. Several factors were individually assessed for their power to predict adaptation magnitude (calculated as in Figure 2B) using linear regression: (1) mean (retinal) error, (2) mean corrective saccade amplitude, (3) ISS – σ_b_, (4) ISS – “inherent hypometria” (IH), (5) mean error – IH, and (6) ISS (Figure 5). We used the first 25 trials of the adapt phase to compute mean error (relative to the target’s location after ISS) and corrective saccade amplitude, since both change quite rapidly during adaptation. The term “inherent hypometria” is taken from *Wong and Shelhammer* who found that the best predictor of adaptation was the difference between error and IH [15]; we calculated IH as the average difference between target location and primary saccade end-point, in the final 50 trials of the baseline. Note that in these plots, we have used degrees of visual angle on the abscissa and gain-units on the ordinate.

We found ISS to have the largest R^2^ (0.77, p<<0.01) of any factor examined (Figure 5). Interestingly, mean error – IH had the smallest R^2^ (0.01, p = 0.49). Note also that while factors (3) and (4) both had large, significant R^2^ values (0.68, and 0.76, respectively, both p<<0.01), both were decrements in explanatory power relative to ISS alone. The finding that ISS has the greatest ability (of any individual factor) to explain adaptation is consistent both with the notion that ISS best accounts for adaptation in conventional (consistent ISS) adaptation paradigms, and with our contention that end-point variability does not determine ISS sensitivity. However, this result does not rule out the possibility that factors other than ISS simply play a lesser or more modulatory role in adaptation.

A stepwise linear regression was conducted to determine whether a combination of factors might best account for adaptation magnitude. Only ISS was necessary to explain the amount of observed adaptation (regression coefficient: 0.76, t-stat: 10.69, p<<0.01), and no additional explanatory power was furnished by any of the remaining terms, which were: (1) ISS size, (2) “inherent hypometria” (IH), (3) mean (retinal) error, (4) mean corrective saccade amplitude, (5) σ_b_ (baseline variability), and (6) ISS/σ_b_. The final factor was included explicitly to test whether ISS sensitivity might depend on endpoint variability in a divisive (non-linear) fashion; while regression analysis is capable of revealing additive (linear) relationships amongst predictive factors, it cannot capture such divisive relationships unless they are considered directly as we have done. That is, this ratio of factors was included to ensure we were considering a wide variety of putative predictors of adaptation. The results of this stepwise regression suggest that adaptation magnitude depends most directly on ISS size; that the other factors that we considered do not meaningfully improve the ability to predict the magnitude of adaptation, or that these other factors contribute to the adaptation process in a nonlinear fashion which we have not considered.

### Recovery

Significant recovery occurred whenever significant adaptation had occurred (Figure 2C). Similar to the completeness of adaptation, across subjects, we only observed complete recovery in the 0.025-ISS condition (Figure 2C; recovery magnitude was not significantly different from the magnitude of adaptation; α = 0.05). Recovery appeared to saturate to a greater extent than did adaptation: despite significantly greater adaptation in the 0.1-ISS compared to the 0.075-ISS condition (Figure 2B; α = 0.05), recovery magnitudes were indistinguishable (α = 0.05).

## Discussion

Our results are nicely in keeping with the one prior study (of which we are aware) that systematically varied ISS size. That study observed that backwards ISSs ranging from 25–50% of the primary target step resulted in proportional gain decreases that reached 60% completeness [16]. Our ISSs ranged from 0–10% of the primary target step, and gain changes reached 70% completeness (on average). Interestingly, measuring adaptation as a proportion of ISS revealed that a constant percent-completeness was achieved across conditions. This is in contrast to adaptation studies using fixed errors [12], as well as other motor learning work [25], [28], which have found that smaller errors may be treated differently than large.

The most notable feature of our results is the finding that a roughly constant proportion of ISS is compensated for across conditions. This finding is in stark contrast with the hypothesis that saccade adaptation is driven by retinal error: if adaptation halted once a retinal error goal was reached, the proportion of ISS achieved would increase with ISS; if anything, our results hint at the opposite trend (Figure 3D). Further work will be required to determine whether our finding and those of *Miller et al. –* of a roughly constant percent completeness –generalize to a variety of primary target step amplitudes and other conditions [16].

This present study is part of a growing literature that suggests retinal error is not the primary signal driving saccade adaptation in humans. We found that ISS was the best predictor of adaptation magnitude, and that the additional factors we considered – retinal error amongst them – did not improve that prediction. Several other studies have highlighted examples where retinal error is a poor predictor of adaptation as well. *Bahcall and Kowler* asked subjects to purposefully make saccades partway to a target that was stepped intrasaccadically [29]. Despite large positive retinal errors, this paradigm led to a decrease in saccade gain. As previously noted, *Wong and Shelhammer* found that retinal error did not account for the adaptation they observed in their fixed post-saccadic retinal error study [15]. Meanwhile, work from our own lab demonstrates that even when the specific trial-to-trial pattern of retinal errors from one session is exactly reproduced in a subsequent session (with the same subject), but with different ISSs, the magnitude of adaptation differs significantly [30]. All of these studies suggest that a “prediction error” (the difference between expected and actual post-saccadic target location), drives the adaptation.

If a prediction error does indeed drive saccade adaptation, our results suggest that the predictive mechanism can be quite accurate. We found significant adaptation across subjects with an ISS of 0.025, smaller than any individual’s σ_b_ (baseline saccade endpoint variability) in any session (Figure 4); as well as several examples of individuals who adapted to 0.05 ISSs despite having σ_b_ >0.05. This implies that predictions of post saccadic target location must be at least as accurate as the ISS size. In keeping with this conclusion are results from the study of two-saccade sequences. When the second saccade in such a sequence is memory guided, and no visual landmark is available at the conclusion of the first saccade, making an accurate second saccade requires compensation for the natural variability in landing position arising from the first saccade. Several works have explored the nature of such compensation [31]–[34] with differing findings regarding its extent. However, it appears that in many cases, compensation can be complete [33], [34]. Thus, it is not unreasonable to suggest that individuals can, on a trial-by-trial basis, detect deviations from prediction of the same scale as our minimum effective ISSs.

As the primary goal of the present work was to explore the effect of systematic variation of ISS amplitude on adaptation, we have chosen to largely ignore the question of how recovery from adaptation might be affected by adaptation with such small ISSs. However, visual inspection of our recovery data (Figure 3C) indicates that they likely do not follow the same proportion trend as our adaptation data, suggesting that further work on this front may be fruitful.

Our results also leave open the question of what accounts for inter-subject differences in minimum effective ISS. That such differences were present in our data suggests that individuals either possess differing capacities for detecting intrasaccadic perturbations, or differing capacities to adaptively respond to them. Unfortunately, the present results do not allow us to disambiguate these two possibilities. It is worth noting however, that both are consistent with previously mentioned results from two-saccade sequence experiments, in which it is apparent that the ability to compensate in the second-saccade for variability in first-saccade differs amongst individuals [31], [34]. Further work will be required to clarify this important distinction.

In conclusion, our results support the notion that, when of fixed size, intrasaccadic target steps are a better predictor of adaptation magnitude than retinal error. In addition, our results suggest that one cannot equate saccade end-point variability with “noise” in the error signal for adaptation. Rather, it seems that the oculomotor system may use a non-visual estimate of saccade end-point which allows the estimation of saccadic error to be much more accurate than previously appreciated.
